# Lack of multiple paternity in the oceanodromous tiger shark (*Galeocerdo cuvier*)

**DOI:** 10.1098/rsos.171385

**Published:** 2018-01-17

**Authors:** Bonnie J. Holmes, Lisa C. Pope, Samuel M. Williams, Ian R. Tibbetts, Mike B. Bennett, Jennifer R. Ovenden

**Affiliations:** 1 School of Biomedical Sciencesy, The University of Queensland, St Lucia, Queensland 4072, Australia; 2 Molecular Fisheries Laboratory, The University of Queensland, St Lucia, Queensland 4072, Australia; 3 School of Biological Sciences, The University of Queensland, St Lucia, Queensland 4072, Australia; 4 Department of Agriculture and Fisheries, Brisbane, Queensland 4001, Australia; 5 Institute for Social Science Research, The University of Queensland, Long Pocket Precinct, Queensland 4072, Australia

**Keywords:** elasmobranch, aplacental viviparity, multiple paternity, polyandry

## Abstract

Multiple paternity has been documented as a reproductive strategy in both viviparous and ovoviviparous elasmobranchs, leading to the assumption that multiple mating may be ubiquitous in these fishes. However, with the majority of studies conducted on coastal and nearshore elasmobranchs that often form mating aggregations, parallel studies on pelagic, semi-solitary species are lacking. The tiger shark (*Galeocerdo cuvier*) is a large pelagic shark that has an aplacental viviparous reproductive mode which is unique among the carcharhinids. A total of 112 pups from four pregnant sharks were genotyped at nine microsatellite loci to assess the possibility of multiple paternity or polyandrous behaviour by female tiger sharks. Only a single pup provided evidence of possible multiple paternity, but with only seven of the nine loci amplifying for this individual, results were inconclusive. In summary, it appears that the tiger sharks sampled in this study were genetically monogamous. These findings may have implications for the genetic diversity and future sustainability of this population.

## 1. Introduction

Increased human exploitation over the past two decades coupled with increasing habitat modification poses immediate threats to shark populations worldwide [1]. Many species of sharks have low resilience to exploitation because of their life-history characteristics, such as late age maturity and low fecundity [2,3]. Coupled with the demand for shark-related product driving the depletion of shark populations worldwide, the removal of oceanic predators is predicted to have serious consequences for entire marine ecosystems [4]. To adequately understand the implications of these findings, the examination of life-history parameters is crucial to determine the extent a species' population would be affected by fishing and the ability to recover if stocks become depleted [5,6]. Apart from assessing stock structure and life-history traits such as age and growth, it has become apparent that data on mating systems are paramount in order to monitor shark populations more accurately [7–10]. While the reproductive structures and functions of elasmobranchs are quite well characterized [11], the relationships between ecology, reproductive mode and reproductive strategies are less understood [12].

Multiple mating by females is referred to as polyandry and is one mating strategy widely documented to increase the genetic quality of offspring [8,13]. Theoretically, certain genes or genetic combinations will raise the mean offspring fitness of polyandrous females, compared with that obtained from a single mating [13]. Multiple paternity occurs when a single brood of offspring is fertilized by multiple males [9], and has been documented in both viviparous and ovoviviparous elasmobranchs (e.g. [14]) leading to the assumption that multiple paternity may actually be ubiquitous in cartilaginous fishes [10,12].

The majority of studies assessing elasmobranch multiple paternity have been conducted on coastal and nearshore species, with a lack of research on pelagic sharks, almost certainly due to the inaccessibility of samples [12]. In the case of large oceanic species, obtaining pups from females of reproductive age remains challenging, particularly when aborting of pups is commonplace during capture [15]. In addition, the difficulty of observing mating events in species that do not aggregate negates direct behavioural observations [16]. Genetic studies on polyandry and multiple paternity enable patterns of reproductive behaviour to be inferred by using bi-parentally inherited nuclear DNA markers, such as microsatellites. By comparing the paternal genetic contribution to each of the offspring in a litter, multiple paternity has been identified in elasmobranchs using as few as four microsatellites (see [14], for review). In one of the few studies on oceanic sharks, Corrigan *et al*. [12] reported multiple paternity in the shortfin mako (*Isurus oxyrinchus*) using just one litter and five microsatellite loci. Concomitantly, Gubili [17] used seven polymorphic microsatellite loci on one litter of great white sharks (*Carcharodon carcharias*), and also found evidence of multiple sires.

The tiger shark (*Galeocerdo cuvier*) (Péron and Lesueur 1822) is the largest species in the family Carcharhinidae, with a circumglobal distribution in both tropical and warm temperate neritic and pelagic waters. Off the Australian east coast, *G. cuvier* maintains variable home ranges, with movements extending across the broader Indo-West Pacific into both tropical and seasonally warm temperate waters [18]. Throughout the region, *G. cuvier* is targeted primarily by recreational game fishers, shark control programmes and commercial fishing operations [19–21]. Estimates of the relative susceptibility of tiger sharks off the east coast of Australia revealed that the species ranks as one of the highest to risk from commercial fishing operations [22]. The large size, semi-solitary nature and the wide-ranging movements of this species have, thus far, hindered a comprehensive study of its biology, which is essential for the development of appropriate management strategies. With the capacity to rebound from population reductions often directly linked to the reproductive potential of a species, understanding a species’ reproductive strategies is vital for effective fisheries management and conservation. To date, the management arrangements for the species in Australian waters were developed using a precautionary approach given the paucity of information regarding its biology and general life-history characteristics.

Tiger sharks are the only carcharhinid with an aplacental viviparous (ovoviviparous) reproductive strategy [15], making them unique within the order Carcharhiniformes. While reproductive data on this species from Australian waters remain scant, selected aspects of tiger shark reproductive biology including size at maturity, gestation period, timing of parturition, and size at birth have been investigated [15,23–32]. Regional differences in female reproductive cycle have also been reported, with biennial cycles reported in Atlantic tiger sharks [31], and triennial cycles in tiger sharks sampled from the Pacific Ocean [15]. While long-term sperm storage in the oviducal glands of tiger sharks has been identified [33,34], it remains unknown as to whether sperm from different males can be stored. As with other migratory sharks that employ polyandry as a reproductive strategy (e.g. *C. carcharias* [17] and *I. oxyrinchus* [12]), we hypothesize that female tiger sharks will also mate with multiple males and produce litters with multiple sires. Here, we examine four litters of *G. cuvier* pups from northeast Australian waters to investigate the presence of multiple paternity in this species for the first time.

## 2. Material and methods

**Figure 1.**
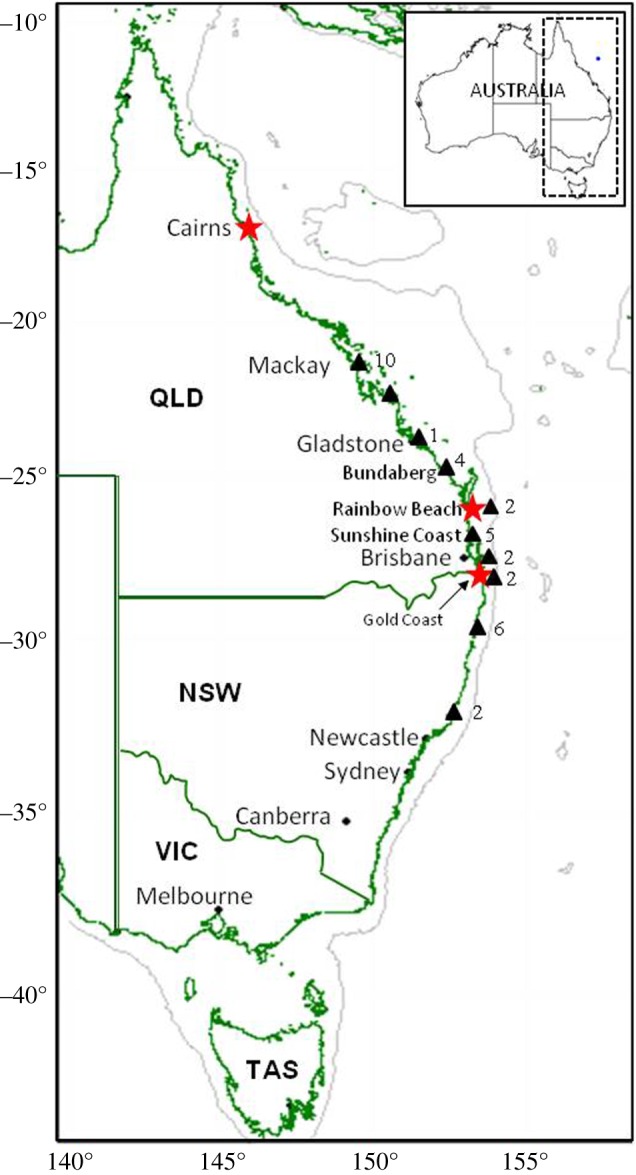
Capture locations of *Galeocerdo cuvier* mother–litter groups (*n* = 4, red stars), and population samples used to determine allele frequencies (*n* = 34, black triangles) from the Australian east coast. Numbers beside black triangles indicate sample sizes from that location. Note two mother–litters were captured at Rainbow Beach.

Genotypes were obtained from the fin clips of 112 embryos from four litters (63 female, 49 male) captured in the Queensland Shark Control Program (QSCP) between 2008 and 2012 [35]. The pregnant sharks were captured on drumlines at Rainbow Beach (2 litters), Gold Coast (1 litter) and Cairns (1 litter; figure 1). To the best of our knowledge, full litters were received from the Gold Coast and Rainbow Beach females (litter sizes 26–36); however, only 16 pups from a litter of 62 were made available to this study from the shark contractor in Cairns (table 1). To test for multiple paternity among pups, nine microsatellite loci developed for tiger sharks were used [36]. The maternal genotype was not collected for one litter. Tissue was stored in 95% ethanol until laboratory processing. DNA extraction was performed using either a QIAGEN DNeasy blood and tissue extraction kit following the manufacturer's protocols (QIAGEN Inc., Valencia, CA), or a salting out method [37]. PCR amplification of loci was optimized using the system reported in [38]. To estimate allele frequencies for the local population, genotyping was completed for 34 adults randomly sampled from a collection of several hundred individuals previously captured (described in [38]). Departure from Hardy--Weinberg equilibrium was tested using the Markov chain method in GENEPOP 4.1.3 [39], with 100 000 dememorization steps, 100 batches and 10 000 subsequent iterations. The number of alleles, unbiased expected heterozygosity and the probability of identity of siblings (PIDsib) were estimated using GenAlEx 6.5 [40]. Genotypes were checked for null alleles and scoring errors using Micro-Checker 2.2.3 [41]. We also tested for linkage disequilibrium among loci using an exact test based on a Markov chain method as implemented in GENEPOP, in both cases using sequential Bonferroni to correct for multiple tests (*p* < 0.05; as per [38]).

**Table 1.** *Galeocerdo cuvier* litter characteristics including date and location of capture, litter size, sex ratio (female:male), mother and mean pup size ± s.e. (centimetres (cm), and total length (TL)). Sex ratio for Litter 4 (partial litter) not calculated as equal numbers of males and females were provided to the study.

*Litter 1*	
Capture date	26 Dec 2008
Litter size	26
Location	Rainbow Beach
Sex ratio F:M	1.36
Mother size (cm TL)	450
Mean pup size (cm TL) ± s.e.	74.15 ± 0.35
*Litter 2*	
Capture date	12 Oct 2011
Litter size	34
Location	Gold Coast
Sex ratio F:M	2.09
Mother size (cm TL)	380
Mean pup size (cm TL) ± s.e.	71.22 ± 8.8
*Litter 3*	
Capture date	05 Dec 2011
Litter size	36
Location	Rainbow Beach
Sex ratio F:M	0.89
Mother size (cm TL)	370
Mean pup size (cm TL) ± s.e.	69.3 ± 0.4
*Litter 4*	
Capture date	04 Aug 2012
Litter size	16
Location	Cairns
Sex ratio F:M	n.a.
Mother size (cm TL)	450
Mean pup size (cm TL) ± s.e.	57.9 ± 0.9

To determine the power for detecting multiple paternity, we performed simulations using PrDM 1 [42]. This software calculates the probability of detecting multiple sires given: (i) allele frequencies in the adult population; (ii) differing litter sizes and (iii) differing multiple paternity rates. We simulated three multiple paternity scenarios: (i) two fathers’ equal paternity (50% each); (ii) two fathers’ moderate skew (66%, 33%) and (iii) two fathers’ high skew (92.5%, 7.5%). The ‘high skew’ scenario represents approximately one pup with a different father in a litter of 15. Simulations were performed across the range of litter sizes (16–36) in our study, using allele frequencies estimated from the Australian east coast population (34 adults) and assuming maternity was unknown.

Paternity of litters was determined using two methods: manual allele counting (see also [7,43,44]), and a full pedigree likelihood method, executed in Colony 2.0.5.5 [45,46]. Manual allele counting was undertaken by subtracting the maternal alleles and identifying the number of unique paternal alleles at each locus. The total number of alleles per locus for each litter was also quantified. The presence of more than two paternal alleles across at least two loci was considered evidence for multiple paternity [7]. The software Colony 2.0.5.5 infers sibships and parentage based on multilocus genotypes by assigning offspring to full- or half-sib families. Pedigrees for each cluster are constructed by the software, and then pedigree likelihoods are compared to define sibling groups. Maternal genotype was included where known (assigned with high confidence, 0.999), and a low uniform error rate was applied (0.0001).

## 3. Results

Preliminary screening of adult genotypes from selected east coast Australian locations detected no significant deviation from Hardy–Weinberg disequilibrium, and all locus pairs were in linkage equilibrium following sequential Bonferroni correction (*p* < 0.05). Mendelian inheritance of alleles at these loci was further supported by the complete concordance of mother–offspring genotypes (112 comparisons). The nine loci had an average of 10.3 alleles (range 3–22) and unbiased heterozygosity of 0.72 (range 0.43–0.93; table 2). This allowed differentiation of siblings with high confidence (PIDsib = 0.0003).

**Table 2.** Microsatellite marker diversity for east Australian *G. cuvier* (*n* = 34) described by number of alleles (*N*_a_), effective number of alleles (*N*_e_), observed (*H*_o_), unbiased expected heterozygosity (u*H*_e_), expected heterozygosity (*H*_e_) and inbreeding coefficient (*F*_is_).

	*Locus*								
	Tgr_1033	Tgr_1157	Tgr_1185	Tgr_212	Tgr_233	Tgr_348	Tgr_47	Tgr_891	Tgr_943
*N*_a_	4	8	6	3	22	16	5	18	11
*N*_e_	1.903	5.415	3.446	1.783	8.377	9.553	1.737	11.446	7.945
*H*_o_	0.353	0.735	0.706	0.382	0.941	0.939	0.441	0.882	0.853
u*H*_e_	0.482	0.827	0.72	0.446	0.894	0.909	0.431	0.926	0.887
*H*_e_	0.474	0.815	0.71	0.439	0.881	0.895	0.424	0.913	0.874
*F_is_*	0.256	0.098	0.005	0.129	−0.069	−0.049	−0.04	0.033	0.024

Manual allele counting indicated that only one father contributed to each litter. None of the three litters where maternity was known had more than two paternal alleles for at least two loci. For the litter where the mother was not known, the total allele count per locus in the litter did not exceed 4, which implied a single sire. If two fathers contributed equally to litters, or with only moderate skew (66 : 33%), PrDM simulations indicated that we had strong power to detect multiple paternity, even for the smallest litter size (*n* = 16, *p* > 0.999; table 3). However, detecting multiple paternity at high skew was more difficult (92.5 : 7.5%; e.g. approx. 1 offspring out of 13 with a second father). The smallest litter size had a probability of 0.71 of detecting high skew, but for the largest litter this increased to 0.939, indicating that in all but the most extremely skewed scenarios, evidence of multiple paternity would have been detected (table 3).

**Table 3.** Probability of detecting multiple paternity for the nine microsatellite loci used under three scenarios varying in number of paternal skews.

	number of embryos				
	16	20	25	30	36
paternal skews					
two males (50 : 50)	1	1	1	1	1
two males (66.7 : 33.3)	0.999	1	1	1	1
two males (92.5 : 7.5)	0.713	0.79	0.858	0.902	0.939

**Figure 2.**
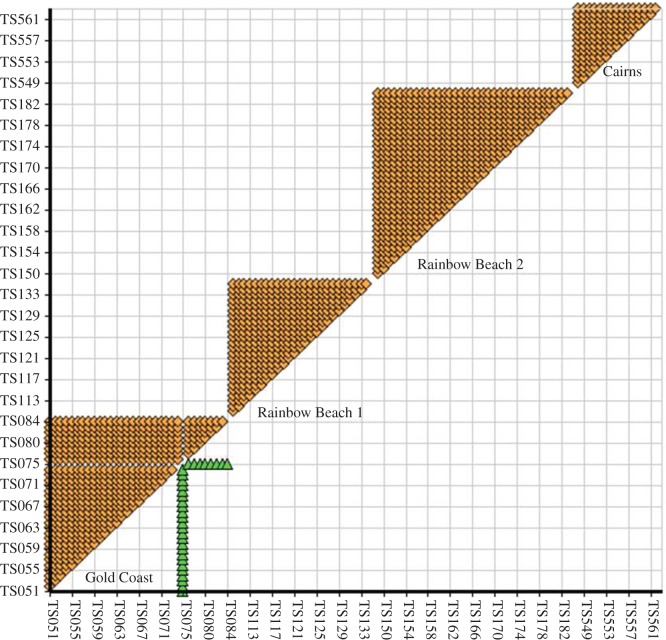
Sibling relationships (sibships) based on pedigrees for each litter, indicated by yellow diamonds. Three of the four litters are fathered by a single male. A single pup (TS075; green triangles) is proposed as a half-sibling, and assigned to a second father (probability of substructure = 0.999).

The manual allele counting method to estimate the number of fathers was confirmed using the software Colony. Three litters were identified by Colony as being fathered by a single male. In the fourth litter, a single pup, out of a litter of 34, was assigned to a second father with high confidence (probability of substructure = 0.999; figure 2). The second male assignment was based on two loci; however, the pup was homozygous at these loci with an allele that matched its mother. Thus, while it is possible that this pup was the result of a second male gaining paternity, this assignment could also be due to allelic dropout. Only seven of the nine loci could be amplified for this pup, and repeated genotyping was unsuccessful.

## 4. Discussion

This study provides the first genetic assessment of the reproductive strategy of *G. cuvier*, highlighting a potentially critical aspect of the species' life history. Successful mating in sharks with wide-ranging coastal and pelagic movements may depend upon the rate of encounter between potential mates [9], and because of this, multiple mating is probably less common [47]. This may be further compounded when the stock is impacted by fishing exploitation, decreasing the rate of encounter due to declines in abundance or changes in sex ratios of mature individuals [9]. The capacity to rebound from population reductions is often directly linked to the reproductive potential of a species; therefore, the knowledge of species-specific reproductive strategies is central to the development of appropriate management and conservation plans [48].

Despite genotyping 112 pups from four different litters, evidence for multiple paternity (more than two paternal alleles, more than one offspring assigned to a second male) in tiger sharks from this population was limited to potentially one individual. Instead, the data indicate predominantly single-sired litters in the tiger sharks sampled herein. If multiple paternity does occur in tiger sharks in this population, it does so at extremely low frequencies within litters (1/34; in 1 of 4 litters). Although multiple paternity is widely accepted as a common reproductive strategy in elasmobranchs, the frequency and prevalence may vary between species, populations and even between conspecific individuals [9,49].

Genetic monogamy has been reported in only one other elasmobranch, the bonnethead shark (*Sphyrna tiburo*), with 22 litters sampled from the Gulf of Mexico revealing over 80% as being single-sired [7]. Most notable was the presence of multiple paternity only in mothers that were significantly larger and had more offspring than mothers of single paternity litters. It was noted by Chapman *et al*. [7] that species with large, highly dispersive populations probably have lower levels of polyandrous mating and multiple paternity than those with small, fragmented or less dispersive populations. Given there remains only scant evidence of philopatry to mating and pupping grounds for *G. cuvier* [50], and that there is little population differentiation across the Indo-West Pacific region [38], it appears that like *S. tiburo*, tiger sharks are either predominantly monogamous (males and females producing offspring with a single partner each reproductive cycle), or polygynous (males producing offspring with multiple females, but females only producing offspring with one male [7]).

Given the semi-solitary ecology of *G. cuvier*, Pratt [34] proposed that the duration of long-term storage of viable sperm in the female oviducal gland would match at least the gestation period for the species, with repeat fresh inseminations required to increase the chance of fertilization in the absence of male contact. The lack of multiple paternity found in this study may indicate that sperm is either stored from a single male at a time, or, in the event of multiple matings, some other form of post-copulatory process is taking place. Reported widely in other taxa, physiological mechanisms such as sperm competition [51], sperm ‘flushing’ [52], and facilitating or inhibiting sperm mixing in the oviducal gland prior to fertilization [53] remains unknown in chondrichthyan fishes. Comparative studies on oviducal gland morphology with the prevalence of multiple paternity will significantly increase our understanding of how cryptic female choice might occur in elasmobranchs [16]. Advances in collection techniques may also allow researchers to directly genotype stored sperm, allowing for comparisons between successful matings and the genotypes observed in resultant litters.

Although only four litters were analysed in this study, several other studies analysing between one and four litters discovered multiple paternity in a range of sharks, including species that also employ an aplacental reproductive strategy (e.g. *C. altimus*, *n* = 1 [9]; *Hexanchus griseus*, *n* = 1 [54]; *Ginglymostoma cirratum*, *n* = 3 [55]; *Isurus oxyrinchus*, *n* = 4 [56] and *n* = 1 [12]; *C. carcharias*, *n* = 1 [56]). Furthermore, the large litter sizes tested for paternity in this study when compared with other studies of smaller litter sizes [14,57] should have increased the chance of discovering multiple paternity across a litter, even at low frequencies. The presence of a single pup with two homozygous loci that potentially had a different sire was inconclusive, based on only 7 of the 9 microsatellite loci amplifying, and the repeated genotyping three times failed to validate the original results due to degraded DNA.

With some studies suggesting that multiple paternity may maintain genetic variation in a population, or increase effective population size [58,59], there is a greater likelihood that some of the offspring in a litter will be more adaptive to changing environmental conditions [57,60]. This might be particularly relevant in elasmobranchs, which generally exhibit a slower rate of molecular evolution than other vertebrates [57]. Compared with sharks that employ multiple paternity as a mating strategy, the effective population size of *G. cuvier* may be strongly constrained by the total number of breeding females [58].

Understanding the reproductive strategies of commercially and recreationally exploited elasmobranchs is fundamental to implementing appropriate fisheries management regimes. The lack of evidence supporting multiple paternity in this species may indicate that tiger shark populations are more vulnerable to the loss of genetic diversity than other sharks which use this strategy. With most females not sexually mature until approximately 325 cm TL (approx. 12 years of age; [32]), coupled with a possible triennial breeding cycle decreasing annual fecundity to around 33% [15], tiger sharks in this region may have a reduced capacity to withstand significant amounts of fishing pressure. Together with the recent catch rate declines identified on the Australian east coast [21], additional management measures to ensure the sustainability of tiger sharks in this region may be required.

## Data accessibility

Tiger shark population genotypes are available through the UQ eSpace at http://espace.library.uq.edu.au/view/UQ:613874 and the mother and pup genotypes are available at https://espace.library.uq.edu.au/view/UQ:696676.

## References

[1] Cortés E 2000 Life history patterns and correlations in sharks. Revs Fish. Sci. 8, 299–344. (doi:10.1080/10408340308951115)

[2] Musick JA, Burgess G, Cailliet G, Camhi M, Fordham S 2000 Management of sharks and their relatives (Elasmobranchii). Fisheries 25, 9–13. (doi:10.1577/1548-8446(2000)025<0009:MOSATR>2.0.CO;2)

[3] Ferretti F, Myers RA, Serena F, Lotze HK 2008 Loss of large predatory sharks from the Mediterranean Sea. Conserv. Biol. 22, 952–964. (doi:10.1111/j.1523-1739.2008.00938.x) 18544092 10.1111/j.1523-1739.2008.00938.x

[4] Myers RA, Worm B 2003 Rapid worldwide depletion of predatory fish communities. Nature 423, 280–283. (doi:10.1038/nature01610) 12748640 10.1038/nature01610

[5] Branstetter S 1990 Early life-history implications of selected carcharhinoid and lamnoid sharks of the northwest Atlantic. In Elasmobranchs as living resources: advances in the biology, ecology, systematics, and the status of the fisheries (eds Pratt HL, Gruber SH, Taniuchi T), pp. 17–28. NOAA Technical Report NMFS 90. See https://spo.nmfs.noaa.gov/tr90opt.pdf.

[6] Smith SE, Au DW, Show C 1998 Intrinsic rebound potentials of 26 species of Pacific sharks. Mar. Fresh. Res. 49, 663–678. (doi:10.1071/MF97135)

[7] Chapman DD, Prodohl PA, Gelsleichter J, Manire CA, Shivji MS 2004 Predominance of genetic monogamy by females in a hammerhead shark, *Sphyrna tiburo*: implications for shark conservation. Mol. Ecol. 13, 1965–1974. (doi:10.1111/j.1365-294X.2004.02178.x) 15189217 10.1111/j.1365-294X.2004.02178.x

[8] Neff BD, Pitcher TE 2005 Genetic quality and sexual selection: an integrated framework for good genes and compatible genes. Mol. Ecol. 14, 19–38. (doi:10.1111/j.1365-294X.2004.02395.x) 15643948 10.1111/j.1365-294X.2004.02395.x

[9] Daly-Engel TS, Grubbs RD, Holland KN, Toonen RJ, Bowen BW 2006 Assessment of multiple paternity in single litters from three species of carcharhinid sharks in Hawaii. Env. Biol. Fish. 76, 419–424. (doi:10.1007/s10641-006-9008-5)

[10] Portnoy D, Heist EJ 2012 Molecular markers: progress and prospects for understanding reproductive ecology in elasmobranchs. J. Fish Biol. 80, 1120–1140. (doi:10.1111/j.1095-8649.2011.03206.x) 22497375 10.1111/j.1095-8649.2011.03206.x

[11] Conrath CL, Musick J 2012 Reproductive biology of elasmobranchs. In Biology of sharks and their relatives (eds Carrier J et al.), pp. 291–392. Boca Raton, FL: CRC Press.

[12] Corrigan C, Kacev D, Werry JM 2015 A case of genetic polyandry in the shortfin mako *Isurus oxyrinchus*. J. Fish Biol. 87, 794–798. (doi:10.1111/jfb.12743) 26219624 10.1111/jfb.12743

[13] Jennions MD, Petrie M 2000 Why do females mate multiply? A review of the genetic benefits. Biol. Revs. 75, 21–64. (doi:10.1111/j.1469-185X.1999.tb00040) 10740892 10.1017/s0006323199005423

[14] Rossouw C, Wintner SP, Bester-van der Merwe AE 2016 Assessing multiple paternity in three commercially exploited shark species: *Mustelus mustelus*, *Carcharhinus obscurus* and *Sphyrna lewini*. J. Fish Biol. 89, 1125–1141. (doi:10.1111/jfb.12996) 27237109 10.1111/jfb.12996

[15] Whitney NM, Crow GL 2007 Reproductive biology of the tiger shark (*Galeocerdo cuvier*) in Hawaii. Mar. Biol. 151, 63–70. (doi:10.1007/s00227-006-0476-0)

[16] Fitzpatrick JL, Kempster RM, Daly-Engel TS, Collin SP, Evans JP 2012 Assessing the potential for post-copulatory sexual selection in elasmobranchs. J. Fish Biol. 80, 1141–1158. (doi:10.1111/j.1095-2012.03256x) 22497376 10.1111/j.1095-8649.2012.03256.x PMC3842027

[17] Gubili C 2008 Application of molecular genetics for conservation of the great white shark, *Carcharodon carcharias*. PhD thesis, School of Biological Sciences, Aberdeen University, UK.

[18] Holmes BJ, Pepperell JP, Griffiths SP, Jaine FRA, Tibbetts IR, Bennett MB 2014 Tiger shark (*Galeocerdo cuvier*) movement patterns and habitat use determined by satellite tagging in eastern Australian waters. Mar. Biol. 161, 2645–2658. (doi:10.1007/s00227-014-2536-1)

[19] Pepperell JG 1992 Trends in the distribution, species composition and size of sharks caught by gamefish anglers off south-eastern Australia, 1961–90. Aus. J. Mar. Fresh. Res. 43, 213–225. (doi:10.1071/MF9920213)

[20] Macbeth WG, Geraghty PT, Peddemors VM, Gray CA 2009 Observer-based study of targeted commercial fishing for large shark species in waters off nothern New South Wales. NSW, Australia: Industry and Investment New South Wales.

[21] Holmes BJ, Sumpton WD, Mayer DG, Tibbetts IR, Neil DT, Bennett MB 2012 Declining trends in annual catch rates of the tiger shark (*Galeocerdo cuvier*) in Queensland, Australia. Fish. Res. 129–130, 38–45. (doi:10.1016/j.fishres.2012.06.005)

[22] Gribble NA, Whybird O, Williams L, Garrett R 2005 Fishery assessment update 1988–2003: Queensland east coast shark. Department of Primary Industries and Fisheries, Queensland, Australia Report#QI04070. 26.

[23] Springer S 1938 Notes of the sharks of Florida. Proc. Flor. Acad. Sci. 3, 9–41.

[24] Springer S 1940 The sex ratio and seasonal distribution of some Florida sharks. Copeia 1940, 188–194. (doi:10.2307/1437982)

[25] Fourmanoir P 1961 Requins de la côte ouest de Madagascar. Mem. Ins. Sci. Mad. 4, 1–81.

[26] Clarke E, von Schmidt K 1965 Sharks of the central Gulf coast of Florida. Mar. Sci. Bull. 15, 13–83.

[27] Bass AJ, D'Aubrey JD, Kistnasamy N 1975 Sharks of the east coast of Southern Africa. III. The families Carcharhinidae (excluding Mustelus and Carcharhinus) and sphyrnidae. Durban, South Africa: The Oceanographic Research Institute.

[28] Branstetter S, Musick JA, Colvolcoresses JA 1987 A comparison of the age and growth of the tiger shark, *Galeocerdo cuvier*, from off Virginia and from the northwestern gulf of Mexico. Fish. Bull. 85, 269–279.

[29] Simpfendorfer CA 1992 Biology of tiger sharks (*Galeocerdo cuvier*) caught by the Queensland shark meshing program off Townsville, Australia. Aust. J. Mar. Fresh. Res. 43, 33–43. (doi:10.1071/MF9920033)

[30] Wintner SP, Dudley SFJ 2000 Age and growth estimates for the tiger shark, *Galeocerdo cuvier*, from the east coast of South Africa. Mar. Fresh. Res. 51, 43–53. (doi:10.1071/MF99077)

[31] Castro JI 2009 Observations on the reproductive cycles of some viviparous North American sharks. aqua Int. J. Ichth. 15, 205–222.

[32] Holmes BJ, Peddemors VM, Gutteridge AN, Geraghty PT, Chan RWK, Tibbetts IR, Bennett MB 2015 Age and growth of the tiger shark *Galeocerdo cuvier* off the east coast of Australia. J. Fish Biol. 87, 422–448. (doi:10.1111/jfb.12732) 26248806 10.1111/jfb.12732

[33] Prasad RR 1945 Further observations of the structure and function of the nidamental glands of a few elasmobranchs of the Madras coast. Proc. Ind. Acad. Sci. Sec.B 22, 368–373.

[34] Pratt HL 1993 The storage of spermatozoa in the oviducal glands of western North Atlantic sharks. Env. Biol. Fish. 38, 139–149. (doi:10.1007/BF00842910)

[35] Holmes BJ 2015 The biology and ecology of the tiger shark (*Galeocerdo cuvier*) on the east coast of Australia. PhD thesis, School of Biological Sciences, The University of Queensland, Australia.

[36] Bernard AM, Feldheim KA, Shivji MS 2015 Isolation and characterization of polymorphic microsatellite markers from a globally distributed marine apex predator, the tiger shark (*Galeocerdo cuvier*). Conserv. Gen. Resour. 7, 509–511.

[37] Sunnucks P, Hales DF 1996 Numerous transposed sequences of mitochondrial cytochrome oxidase I-II in aphids of the genus *Sitobion* (Hemiptera: Aphididae). Mol. Biol. Evol. 13, 510–524. (doi:10.1093/oxfordjournals.molbev.a025612) 8742640 10.1093/oxfordjournals.molbev.a025612

[38] Holmes BJ, Williams SM, Otway NM, Nielsen EE, Maher SL, Bennett MB, Ovenden JR 2017 Population structure and connectivity of tiger sharks (*Galeocerdo cuvier*) across the Indo-Pacific Ocean basin. R. Soc. open sci. 4, 170309 (doi:10.1098/rsos.170309) 28791159 10.1098/rsos.170309 PMC5541554

[39] Raymond M, Rousset F 1995 An exact test for population differentiation. Evolution 49, 1280–1283. (doi:10.2307/2410454) 28568523 10.1111/j.1558-5646.1995.tb04456.x

[40] Peakall R, Smouse PE 2012 GenAlEx 6.5: genetic analysis in Excel. Population genetic software for teaching and research—an update. Bioinformatics 28, 2537–2539. (doi:10.1093/bioinformatics/bts460) 22820204 10.1093/bioinformatics/bts460 PMC3463245

[41] Van Oosterhout C, Hutchinson W, Wills D, Shipley P 2004 Microchecker: software for identifying and correcting genotyping errors in microsatellite data. Mol. Ecol. Notes 4, 535–538. (doi:10.1111/j.1471-8286.2004.00684.x)

[42] Neff BD, Pitcher TE 2002 Assessing the statistical power of genetic anlayses to detect multiple mating in fish. J. Fish Biol. 61, 739–750. (doi:10.1111/j.1095-8649.2002.tb00908.x)

[43] Avise JC, Jones AG, Walker D, DeWoody JA 2002 Genetic mating systems and reproductive natural histories of fishes: lessons for ecology and evolution. Ann. Rev. Genet. 36, 19–45. (doi:10.1146/annurev.genet.36.030602.090831) 12429685 10.1146/annurev.genet.36.030602.090831

[44] Hernandez S, Duffy C, Francis MP, Ritchie PA 2014 Evidence for multiple paternity in the school shark *Galeorhinus galeus* found in New Zealand waters. J. Fish Biol. 85, 1739–1745. (doi:10.1111/jfb.12490) 25130757 10.1111/jfb.12490

[45] Wang J 2004 Sibship reconstruction from genetic data with typing errors. Genetics 166, 963–1979. (doi:10.1534/genetics.166.4.1963) 10.1534/genetics.166.4.1963 PMC1470831 15126412

[46] Jones OR, Wang J 2010 COLONY: a program for parentage analysis and sibship inference for multilocus genotype data. Mol. Ecol. Res. 10, 551–555. (doi:10.1111/j.1755-0998.2009.02787.x) 10.1111/j.1755-0998.2009.02787.x 21565056

[47] Gilmore R 1993 Reproductive biology of lamnoid sharks. Env. Biol. Fish. 38, 95–114. (doi:10.1007/BF00842907)

[48] Pirog A, Jaquemet S, Soria M, Magalon H 2017 First evidence of multiple paternity in the bull shark (*Carcharhinus leucas*). Mar. Fresh. Res. 68, 195–201. (doi:10.1071/MF15255)

[49] Boomer JJ, Harcourt RG, Francis MP, Walker TI, Braccini JM, Stow AJ 2013 Frequency of multiple paternity in gummy shark, *Mustelus antarcticus*, and rig, *Mustelus lenticulatus*, and the implications of mate encounter rate, postcopulatory influences, and reproductive modes. J. Hered. 104, 371–379. (doi:10.1093/jhered/est010) 23505312 10.1093/jhered/est010

[50] Bernard AM, Feldheim KA, Heithaus MR, Wintner SP, Wetherbee BM, Shivji MS 2016 Global population genetic dynamics of a highly migratory, apex predator shark. Molecular Ecology 25, 5312–5329. 27662523 10.1111/mec.13845

[51] Birkhead T 2000 Promiscuity: an evolutionary history of sperm competition. Cambridge, MA: Harvard University Press.

[52] Smith RL 1984 Sperm competition and the evolution of animal mating systems. Orlando, FL: Academic Press Inc.

[53] Carrier JC, Pratt HL, Castro JI 2004 Reproductive biology of elasmobranchs. In Biology of sharks and their relatives (eds Carrier JC et al.), pp. 269–286. Boca Raton, FL: CRC Press.

[54] Larson S, Christiansen J, Griffling D, Ashe J, Lowry D, Andrews K 2011 Relatedness and polyandry of sixgill sharks, *Hexanchus griseus*, in an urban estuary. Conserv. Genet. 12, 679–690. (doi:10.1007/s10592-010-0174-9)

[55] Heist EJ, Carrier JC, Pratt HLJ, Pratt TC 2011 Exact enumeration of sires in the polyandrous nurse shark (*Ginglymostoma cirratum*). Copeia 2011, 539–544. (doi:10.1643/ce-10-165)

[56] Gubili C et al. 2012 Application of molecular genetics for conservation of the great white shark, *Carcharhodon carcharias*, L. 1958. In Global perspectives on the biology and life history of the great white shark (ed. Domeier ML). Boca Raton, FL: CRC Press.

[57] Byrne RJ, Avise JC 2012 Genetic mating system of the brown smoothhound shark (*Mustelus henlei*), including a literature review of multiple paternity in other elasmobranch species. Mar. Biol. 159, 749–756. (doi:10.1007/s00227-011-1851-z)

[58] Sugg DW, Chesser RK 1994 Effective population sizes with multiple paternity. Genetics 137, 1147–1155. 7982568 10.1093/genetics/137.4.1147 PMC1206061

[59] Hoekert WEJ, Neufeglise H, Schouten AD, Menken SBJ 2002 Multiple paternity and female-biased mutation at a microsatellite locus in the olive ridley sea turtle (*Lepidochlys olivacea*). Heredity 89, 107–113. (doi:10.1038/sj.hdy.6800103) 12136412 10.1038/sj.hdy.6800103

[60] Yasui Y 1998 The ‘genetic benefits’ of female multiple mating reconsidered. Trend. Ecol. Evol. 13, 246–250. (doi:10.1016/S0169-5347(98)01383-4) 10.1016/s0169-5347(98)01383-4 21238286

